# Building the MCH Public Health Workforce of the Future: A Call to Action from the MCHB Strategic Plan

**DOI:** 10.1007/s10995-022-03377-7

**Published:** 2022-02-16

**Authors:** Lauren Raskin Ramos, Michelle Menser Tissue, Ayanna Johnson, Laura Kavanagh, Michael Warren

**Affiliations:** Maternal and Child Health Bureau, Health Resources and Services Administration, Rockville, MD USA

**Keywords:** Maternal and child health, Public health, Workforce development, Training, Practice

## Abstract

**Introduction:**

In 2021, the Maternal and Child Health Bureau (MCHB) released a new strategic plan to guide its work over the next 10–15 years. The plan highlights four goals—access, equity, workforce capacity, and impact—that are essential to achieving MCHB’s vision.

**Methods:**

We present 13 recommendations to highlight opportunities for ongoing and new activities aligned with Goal 3 of the plan—“*Strengthen Public Health Capacity and Workforce for MCH*.”

**Results:**

Recommendations 1–3 highlight the need to support pathways into state and local MCH public health (PH) positions, to offer accessible and high-quality training for the practicing workforce, and to build capacity to address health and social inequities. Recommendations 4–7 discuss the need to build a racially and ethnically diverse workforce, ensure equity and anti-racism are foundational concepts in training, and strengthen engagement of community members and those with lived experience as part of the MCH PH workforce. Recommendations 8–10 outline opportunities to enhance MCH workforce data and measurement frameworks, and support practice-based research. Recommendations 11–12 discuss the importance of academic-practice partnerships and the need to spur innovation. Recommendation 13 highlights the need to define and amplify the unique skillset of the MCH PH workforce.

**Conclusions:**

The release of the MCHB strategic plan comes at a time of critical need to build and sustain a MCH PH workforce to achieve equity for MCH populations. We encourage the field to engage in dialogue around the recommendations presented in this paper, and to offer additional actions to build and support the MCH PH workforce.

## Significance

*What is already known on this subject?* A highly skilled, diverse MCH workforce is critical to advancing the health and well-being of MCH populations. Yet, the MCH workforce requires ongoing training and support, as well as tailored pathways into academic, state and local MCH public health careers in order to address complex public health issues.

*What this study adds?* This article highlights opportunities for ongoing and new activities to support and strengthen the MCH public health workforce, as aligned with the new MCHB strategic plan goal 3—*Strengthen Public Health Capacity and Workforce for MCH—*and encourages dialogue, partnership, and innovation across the MCH field to build on these recommendations.

## Background/Introduction

**Fig. 1:**
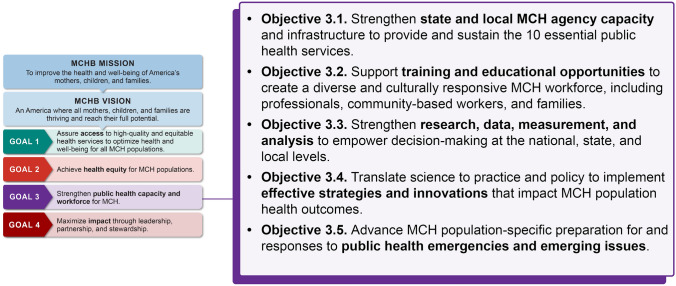
Maternal and Child Health Bureau strategic plan goal 3—strengthen public health capacity and workforce for MCH

In April 2021, the Health Resources and Services Administration’s Maternal and Child Health Bureau (MCHB) released a new strategic plan to guide its work for the next 10–15 years (Health Resources & Services Administration, 2021). The release presents a unique opportunity to consider strategies to build and sustain a MCH public health (PH) workforce that is well-trained, diverse, adequate in size, and prepared to lead complex public health challenges. The strategic plan highlights four goals—access, equity, workforce capacity, and impact—that are essential to achieving MCHB’s vision of an America where all mothers, children and families are thriving and reach their full potential (Health Resources & Services Administration, 2021). While development of the MCH workforce is critical in advancing all four strategic plan goals, this commentary focuses on Goal 3: “*Strengthen Public Health Capacity and Workforce for MCH”* (Fig. 1)—with a specific focus on developing the future and practicing MCH PH workforce. Elevating workforce capacity as a goal of MCHB’s strategic plan affirms the critical role that MCH students, professionals and community members play in advancing the health and well-being of MCH populations.

The COVID-19 pandemic has highlighted the importance of public health infrastructure in protecting and promoting the health of women, children and families. The pandemic placed an unprecedented strain on the nation’s public health workforce, leading to greater workload, burnout, and increased report of mental health conditions (Bryant-Genevier et al., 2021; Stone et al., 2021). These impacts magnified existing challenges for the governmental MCH PH workforce including loss of MCH expertise due to staff attrition, challenges with recruitment and retention of highly skilled staff, lack of racial and ethnic diversity, limited opportunities for advancement, and ongoing barriers to accessing training (Health Resources and Services Administration, Maternal and Child Health Bureau, 2021). Simultaneously, the COVID-19 pandemic intensified inequities for MCH populations that are rooted in the social determinants of health, including discrimination, healthcare access, education, and housing (Centers for Disease Control & Prevention, 2021; Dongarwar et al., 2020).

Over the past ten years, state and local public health agencies have seen a 15 percent decrease in essential staff (de Beaumont Foundation, 2021). A recent analysis of workforce capacity needs indicates that a minimum of 6,500 new full time equivalent MCH staff are needed in state and local public health agencies to provide adequate public health infrastructure and services (de Beaumont Foundation, 2021). Public Health Workforce Interest and Needs Survey (PH WINS) data show that only 14 percent of the state and local governmental MCH workforce has received formal training in public health (de Beaumont Foundation, 2017) and that most employees at state and local health departments are non-Hispanic white, despite continued changes to the racial and ethnic composition of the nation (Sellers et al., 2019).

In Schools and Programs of Public Health (SPPH), challenges persist in demonstrating the critical value of MCH PH education in preparing the future workforce, and in supporting MCH PH faculty. Specifically, there has been a decrease in the number of senior MCH faculty with specialized MCH research, policy, and practice expertise due to retirements (Health Resources & Services Administration, 2019), a consolidation of standalone MCH departments into broader SPPH departments, and pressure to articulate the need for MCH PH graduate education in light of changes to Council on Education for Public Health requirements (Council on Education for Public Health, 2016). These challenges also affect recruitment and retention of postdoctoral fellows and junior faculty pursuing MCH academic career pathways. While 20-year data show increases in the racial and ethnic diversity of public health graduate students and faculty, Black, Hispanic, and Native American faculty continue to be underrepresented among public health faculty positions, particularly as faculty are promoted from assistant- to associate- to full professor (Goodman et al., 2019). Similarly, for students, the admit rate in SPPH for applicants from racial and ethnic groups that are underrepresented (74%) remains lower compared to White and Asian applicants (81%) (Plepys, 2021), highlighting persistent systemic and structural inequities that impact the educational and career trajectories of students from backgrounds that are underrepresented in the public health workforce.

Conversely, the COVID-19 pandemic response prompted innovations in workforce development, an infusion of public health infrastructure support, and increased the visibility of and interest in public health careers, most notably evidenced by a 40% increase in graduate-level applications to SPPH (Association of Schools and Programs of Public Health, 2021). This renewed investment and interest in public health allows us to reimagine MCH PH workforce development. The release of the MCHB strategic plan comes at a historic moment in MCH and public health; a time delineated by both significant challenges and great opportunities to bolster the MCH PH workforce. We must build on this convergence of events to pursue sustainable strategies that support the MCH PH workforce to achieve equity over the next decades.

## Discussion

To catalyze action in response to MCHB’s strategic plan, we highlight opportunities for ongoing and new activities aligned with Goal 3, with a specific focus on the MCH PH workforce. MCHB has historically funded a range of training and workforce development investments to support the current and future MCH workforce in both public health (Table 1) and clinical care.^1^ We acknowledge that the MCHB strategic plan is one of several public health workforce frameworks that provide guidance to the field, and while the following recommendations are not exhaustive, they are unique in their specific focus on the MCH PH workforce. These recommendations cover an array of strategies and will require a coordinated, multisector and multi-organizational approach to advance. We encourage and invite dialogue, partnership, and innovation to build on these recommendations to support the MCH PH workforce of the future.

**Table 1:** Fiscal Year 2021 Maternal and Child Health Bureau/Division of MCH Workforce Development MCH Public Health Workforce Development Investments

Grant program	Target audience	Number of awards	Fiscal year 2021 investment
MCH Leadership, Education, and Advancement in Undergraduate Pathways (LEAP) Training Program	Undergraduate	6	$940,000
MCH Public Health Catalyst Program	Graduate	9	$749,896
Centers of Excellence in MCH Education, Science and Practice MCH Epidemiology Doctoral Training Program Strengthening the MCH Public Health Academic Pipeline	Graduate and post-graduate	13	$5,649,324
MCH Navigator	Practicing professionals	1	$225,000
MCH Workforce Development Center	Practicing professionals	1	$1,720,000

This table denotes MCHB grant programs specific to MCH public health workforce development. MCHB also funds a range of MCH clinical training programs, with a total investment of $45,113,530 in Fiscal Year 2021

Objective 3.1: Strengthen state and local agency capacity and infrastructure to provide and sustain the 10 essential public health services.

*Recommendation 1: Build capacity in state and local MCH agencies to address long-standing, systemic health and social inequities for MCH populations.*

A competent MCH workforce acknowledges and understands the impact of long-standing, systemic inequities and racism on communities and is equipped with the tools to work upstream to address those in partnership with diverse stakeholders. An increasing number of state and local health departments have declared racism a public health crisis and we must ensure the MCH PH workforce has capacity to respond to this crisis (American Public Health Association, 2021; The Network for Public Health Law, 2021). To do so, MCH PH practitioners must gain knowledge and skills to create effective policies and programs aligned with community needs and that dismantle inequities to improve the health of MCH populations (Wysen et al., 2019). Because achieving health equity requires approaches that acknowledge the social, economic and environmental factors that influence health, MCH PH professionals need enhanced skills in areas such as systems transformation and authentic community engagement (Margolis et al, 2017; Raskind et al., 2019; Rivers et al., 2020) and the ability to work across sectors (e.g., housing, transportation, justice, education) to motivate and leverage cross-sector investments to improve MCH.

*Recommendation 2: Develop and support pathways to entry-level career positions in state and local MCH public health, and support career trajectories for MCH staff to enhance job satisfaction and retention.*

Developing a range of pathways to recruit and retain a skilled MCH PH workforce into governmental public health is essential. Strategies can include expanded academic-practice partnerships that lead to employment upon successful completion of an internship, competency-based fellowships, and other public health service opportunities, such as job-sharing with local community organizations (Glynn et al. 2021; Handler et al., 2021). To promote job satisfaction and retention, MCH staff should have developmental opportunities to build new skills, including participation in cross-departmental learning collaboratives, exposure to leaders in the field, job rotations, shadowing, coaching, and mentoring.

*Recommendation 3: Develop and offer accessible, high-quality training, capacity-building, and professional development opportunities for the practicing MCH workforce.*

The MCH PH workforce needs ongoing access to tailored training and capacity building that develops skills aligned to the MCH leadership competencies (Health Resources & Services Administration, 2018). These opportunities should be low or no cost, and provide continued learning in MCH to foster innovation and application of knowledge to real-world challenges. Strategies should also be tailored to practicing professionals, consider the learning needs and styles of non-traditional students (Taylor et al., 2021), be continually assessed for relevance and impact to the field, and adapted to the changing needs of the MCH workforce.

Objective 3.2: Support training and educational opportunities to create a diverse and culturally responsive MCH workforce, including professionals, community-based workers, and families.

*Recommendation 4: Strengthen recruitment and preparation of an MCH public health workforce that is racially and ethnically diverse, and is reflective of the changing demographics across the nation.*

Achieving health equity requires expanded strategies to recruit a diverse MCH PH workforce. Expanding MCH PH training in minority-serving institutions (MSIs), including Historically Black Colleges and Universities, Hispanic Serving Institutions, Tribal Colleges and Universities, and Asian American and Pacific Islander Serving Institutions has the potential to increase the number of students from racially and ethnically diverse backgrounds that enter the MCH PH workforce and that practice in areas that are historically underserved (Noonan et al., 2013). Recruitment and training should target areas with persistent disparities in MCH outcomes to support and encourage students to practice in the communities where they were trained post-graduation. Providing specialized MCH training to community members can also expand the MCH PH workforce and ensure that community members are engaged in addressing persistent MCH challenges.

*Recommendation 5: Ensure equity and antiracism trainings are foundational, grounding concepts in the continuum of MCH training opportunities.*

MCH PH students and practicing professionals require foundational training in equity, the historical roots of inequity, antiracism and cultural responsiveness to effectively work with diverse populations to achieve more equitable outcomes for MCH populations. MCH training and professional development programs have been at the forefront of preparing the MCH PH workforce to tackle complex health challenges, and should continue to develop the workforce to be able to apply upstream approaches that address structural and social determinants that drive inequities in MCH outcomes. In academic programs, this may require support for faculty efforts to strengthen syllabi and program offerings in these areas. Practicing professionals need ongoing training and development in antiracism and systemic inequities, and an equity focus should be integrated into all MCH training opportunities.

*Recommendation 6: Increase exposure to careers in MCH governmental public health for diverse students and professionals.*

Exposure to MCH PH careers that reach students from diverse backgrounds can occur at the high school, undergraduate and graduate levels. Such exposures, which can include a continuum of formal education, mentoring, life skills coaching, cohort-based learning, and paid practica and internship experiences in state and local public health agencies, can have significant impact on the career trajectories and success of students from diverse backgrounds. Other strategies include investing in diversity fellowships for students and early career faculty, branding and marketing MCH career paths to increase visibility of MCH and its impact on public health outcomes, and facilitating partnerships with MSIs, community colleges and community-based organizations to reach diverse students and community members. Investments should target students and community members with varying levels of academic training and lived experience.

*Recommendation 7: Strengthen engagement with MCH-related community organizations, community members, and families as vital members of the MCH workforce.*

The MCH workforce has long recognized the value of partnerships with families, community members and youth to build community capacity to lead and engage as members of the MCH PH workforce. We must continue to amplify the role of families, youth and the community-based workforce (e.g. doulas, home visiting paraprofessionals, community health workers) as part of the MCH PH workforce. Strategies include ongoing training for community members and those with lived experience, and engagement as paid experts, staff and key advisers in the academic and governmental MCH PH workforce. Bolstering capacity of these groups can ensure that they are leaders and partners in developing and implementing public health programs responsive to community needs.

Objective 3.3: Strengthen research, data measurement, and analysis to empower decision-making at the national, state and local levels.

*Recommendation 8: Align and aggregate MCH and public health data sources to understand current workforce composition and highlight current and emerging workforce needs.*

MCH programs in academic, governmental and community contexts draw on available data sources to understand workforce trends and needs, but these sources, when considered individually, lack the nuanced perspective needed to inform a robust understanding of MCH workforce composition and needs. Data sources include enrollment, graduation, and faculty data from academic MCH programs in SPPH, state- and local-based data sources (e.g. PH Workforce Interests and Needs Survey), and data collected through MCHB grant funding (e.g. Title V Information System, MCH Training Programs). Such MCH PH workforce data sources typically focus on only one segment of the workforce (e.g. graduate students, Title V). Thus, there is a need to better document currently available MCH PH workforce data sources and identify how to integrate data to inform a definition of the MCH workforce, identify opportunities for alignment in data collection, and present a comprehensive picture of training and workforce development needs. These efforts can better inform program and policy decisions that influence MCH outcomes.

*Recommendation 9: Develop measurement frameworks that strengthen the link between training and workforce development activities, sustained career pathways in MCH-related fields, and health outcomes for MCH populations.*

There is a need to look beyond current measurement frameworks in order to sustain and increase workforce development investments and effectively communicate the impact of MCH PH training. Multiple methods, including quantitative and qualitative, are needed to formally track graduates of public health education programs to understand the long-term impact of MCH training experiences on career choices and community health outcomes. To assess the impact of investments that build capacity of the practicing MCH workforce, robust data are needed that move beyond changes in knowledge and skill to systematically document and communicate how workforce interventions lead to practice and policy change and the downstream impact on the health of communities and MCH populations.

*Recommendation 10: Expand opportunities for MCH public health faculty and trainees to pursue applied, practice- and policy-focused research.*

MCH PH faculty face challenges in pursuing practice-based research (PBR) including lack of funding and limited institutional support/recognition for PBR as part of the career ladder (Ammerman et al., 2014). To elevate the prestige of PBR, we must fund and incentivize faculty to pursue research aligned to community-identified MCH needs and that promotes shared power between academia, health departments, and community members. SPPH can consider innovative promotion and tenure policies that reward community-engaged research and establish bi-directional opportunities for practitioners and community members to build a culture supportive of practice-oriented research. These opportunities may include adjunct faculty appointments, shared leadership on grant-supported projects, and mentorship/leadership development opportunities for practitioners, students and community members.

Objective 3.4: Translate science to practice and policy to implement effective strategies and innovations that impact MCH population health outcomes.

*Recommendation 11: Formalize and spread models of effective partnerships between state and local MCH agencies and academic MCH public health programs to advance the development and implementation of evidence-based practices.*

State and local health departments, including Title V and MCH public health training programs have a rich history of partnership on research and evaluation, teaching, and practice activities. There is a need to formally document and define the core elements that lead to robust and sustained partnership, and to build capacity among the MCH PH workforce to operationalize these partnerships, especially in light of retirements and ongoing turnover in the MCH PH workforce (Health Resources and Services Administration, Maternal and Child Health Bureau, 2021). Models must consider and elevate the role of community members, youth, and family members in academic-practice partnerships to expand their capacity to participate in planning, analysis, and research practices that center equity and lived experience. Given challenges with budgets and hiring staff at the state and local agency levels, academic-practice partnerships may also fill gaps in knowledge and subject expertise and provide real-time training opportunities for the practicing workforce.

*Recommendation 12: Drive innovation in MCH workforce initiatives by diversifying funding streams and building public–private partnerships to test and replicate new approaches.*

Building an MCH workforce that meets current needs requires exploration of new models of workforce development to build and sustain capacity. Engaging a broad range of funders, including governmental, private and foundation funders is necessary to develop, study, and rapidly spread new, effective models of workforce development. Strategies include learning from and collaborating with other sectors (e.g. business, technology), supporting workforce development innovation and learning labs, using methods such as human centered design to build a workforce responsive to community needs, and expanding use of technology to increase access to MCH training.

Objective 3.5: Advance MCH population-specific preparation for and responses to public health emergencies and emerging issues.

*Recommendation 13: Define and amplify the unique skillset of MCH public health students and practitioners in addressing public health emergencies and emerging issues.*

The COVID-19 pandemic highlighted the need for a workforce that is ready and able to address the complex needs of families and the structural and social determinants that drive inequities. With strength in areas such as life course perspective, systems thinking, social determinants of health, and building and connecting stakeholders, the MCH PH workforce is uniquely poised to anticipate and quickly respond to public health emergencies and emergent issues, and to address associated long-term impacts on MCH populations across the lifespan. We must promote and leverage these strengths across governmental public health to address future public health emergencies and emerging issues. The MCH PH workforce should also be integrated into emergency preparedness activities. MCH PH students and faculty can support a range of emergency response efforts, such as contact tracing, data analysis and research, and can provide MCH subject matter expertise. Academia and the practicing workforce can infuse MCH content and expertise into other public health coursework and with other state and local agencies and PH programs to extend the MCH workforce.

## Conclusion

The release of the MCHB strategic plan comes at a time of critical need to build and sustain a MCH PH workforce that is well-trained, diverse, adequate in size, and prepared to lead efforts to resolve complex public health challenges. There is momentum to invest in public health infrastructure to actively address the impact of the COVID-19 pandemic and the related historic inequities for MCH populations. We must seize these opportunities to elevate the unique strengths of the MCH PH workforce and to demonstrate the critical importance of the MCH PH workforce in achieving equity for MCH populations. We encourage the field to engage in dialogue around the recommendations and strategies presented in this paper, and to offer additional actions to build and support the MCH PH workforce.

## Data Availability

Not applicable.

## Footnotes

1 MCHB workforce development program information can be accessed here: https://mchb.hrsa.gov/training/about-programs.asp.
